# Red Cross and Red Crescent Movement responses to viral hemorrhagic fever outbreaks: A mixed-method evaluation of information management practices for Safe and Dignified Burials

**DOI:** 10.14745/ccdr.v52i78a07

**Published:** 2026-07-30

**Authors:** Douglas Lau, Arwen Barr, Marguerite Gollish, Maria Munoz-Bertrand

**Affiliations:** 1 Canadian Field Epidemiology Program, Centre for Emergency Preparedness, Public Health Agency of Canada, Ottawa, ON; 2 Canadian Red Cross, Ottawa, ON

**Keywords:** Ebola, Marburg, viral hemorrhagic fever, Safe and Dignified Burials, information management, Red Cross and Red Crescent

## Abstract

**Background:**

Ebola and Marburg are viral hemorrhagic fevers (VHFs) with pronounced epidemic potential. Safe and Dignified Burials (SDBs) are a key pillar of humanitarian responses to VHF outbreaks, with Red Cross and Red Crescent (RCRC) National Societies often supporting implementation. The SDB information management (IM) toolkit is a collection of tools and templates that provides guidance for SDB IM across the RCRC Movement.

**Objective:**

To identify lessons learned from the implementation of the SDB IM toolkit and the broader approach to SDB IM in the RCRC Movement, focusing on acceptability, simplicity, and flexibility.

**Methods:**

A sequential mixed-method study design was used, with preliminary findings from key informant interviews guiding development of a subsequent online survey. Procedures for SDB data collection, analysis, and reporting were reviewed, along with how these outputs informed operational decision-making.

**Results:**

Eight interviews and 208 survey responses were analyzed. Acceptability of SDB IM was generally high, though uneven awareness of the SDB IM toolkit and gaps in communicating the strategic value of SDB IM limited broader uptake. Simplicity was viewed favorably, with clearly established workflows and a phased approach to implementation; however, overly detailed guidance documents, limited IM-specific training and insufficient opportunities for hands-on data collection undermined ease of implementation. Flexibility was evident across outbreak settings, though limited analysis and reporting capacity within some National Societies reduced operational adaptability.

**Conclusion:**

While gaps remain, this evaluation highlights the role of SDB IM in supporting data-informed VHF outbreak responses. Strengthening coordination, increasing awareness, expanding IM capacity and supporting greater local ownership may further enhance readiness for future responses.

## Introduction

Ebola virus disease (EVD) and Marburg virus disease (MVD) are severe, often fatal, viral hemorrhagic fevers (VHFs) with pronounced epidemic potential (1). Case fatality ratios are estimated at approximately 50%, though past outbreaks have ranged from 24%–90% (2,3). Unsafe handling of bodies during burials is a well-recognized source of VHF propagation, with studies linking substantial secondary transmission to direct contact with the deceased, their bodily fluids or contaminated materials (2–5). The persistence of VHF outbreaks underscores the ongoing risk to global public health, with nine EVD and seven MVD outbreaks reported to the World Health Organization (WHO) between 2020 and 2025 (2,3,6).

Although the evidence base for Safe and Dignified Burial (SDB) effectiveness is still nascent, several studies have demonstrated their role as critical interventions in reducing transmission (7–9). Consequently, SDBs have become a key pillar in humanitarian responses to VHF outbreaks, with Red Cross and Red Crescent (RCRC) National Societies, supported by the International Federation of Red Cross and Red Crescent Societies (IFRC), taking leadership roles in implementation (10,11).

Routinely collected operational data from SDB activities can support monitoring of outbreak dynamics and intervention outcomes (9); however, maintaining reliable data flows during epidemic scenarios remains challenging (12). These considerations highlight the value of structured SDB information management (IM) in supporting implementation and operational decision-making.

### Safe and Dignified Burial information management

During VHF outbreaks, National Societies often lead SDB implementation, in collaboration with local authorities and other humanitarian partners. While specific processes vary by context, implementation generally follows a similar workflow. When a death meeting epidemiological risk criteria set by local authorities is reported, trained burial teams are dispatched. After obtaining consent, burial teams conduct culturally appropriate burials while maintaining rigorous infection prevention and control measures. Multiple burials may be conducted concurrently across districts or regions, reflecting the scale of outbreaks and corresponding operational demands (13). If additional human resource capacity is required within National Societies, the IFRC surge mechanism facilitates the rapid deployment of trained international delegates to complement existing in-country response capacity (14).

The SDB IM approach is a structured system for collecting, analyzing and utilizing operational data from SDB activities. Under the guidance of an IM focal point, burial team supervisors collect information on the deceased, which is collated and analyzed to generate routine reports, with technical support from the IFRC as needed. These outputs are designed to support situational awareness and are shared with decision-makers to inform operational planning.

To streamline SDB IM implementation, the British Red Cross and IFRC developed the SDB IM toolkit—a package of guidance, tools and templates designed to promote a standardized approach to SDB IM (**Table 1**). Building on lessons learned during the 2013–2015 West Africa EVD outbreak response, the toolkit was formalized in its current form in 2019 and has since been promoted as a framework for SDB IM across RCRC Movement. This evaluation is the first systematic effort to review and refine the approach.

**Table 1:** Safe and Dignified Burial information management toolkit contents

Toolkit component	Description
SDB IM toolkit main guide	Primary guidance document for the SDB IM toolkit. Provides an overview of toolkit components, recommended IM dataflows, activities and timelines; as well as guidance on data protection and information sharing.
Volunteer and staff training guide	Defines human resource requirements and training plans for field implementation of SDB IM.
Data collection guide	Provides templates, key considerations and recommendations for SDB data collection.
Alerts and activities analysis	Introduces a framework for the analysis of SDB outcomes, with key indicators like the percentage of completed burials along with demographic and geographic distributions.
Resistance and failure analysis	Describes a recommended approach to tracking instances of community resistance to SDBs.
Capacity and planning analysis	Specifies key considerations for coordination of large-scale SDB operations with many active burial teams.
Coordination and gap analysis	Gives guidance on the overall coordination of SDB IM and highlights potential gaps to monitor.

Abbreviations: IM, information management; SDB, Safe and Dignified Burial

### Evaluation objective

The overall objective was to identify lessons learned from implementation of the SDB IM toolkit and the broader approach to SDB IM, with a specific focus on the attributes of acceptability, simplicity and flexibility.

## Methods

A sequential mixed-method design was adopted, with preliminary findings from key informant interviews (KIIs) guiding development of a subsequent online survey. Practical experiences with SDB data collection, analysis and reporting were reviewed, along with how resulting insights supported operational decision-making. Data were analyzed and mapped to the attributes of acceptability, simplicity and flexibility, linking findings to the usability and operational value of the SDB IM approach: 1) acceptability was defined as the willingness of RCRC volunteers, staff and delegates to engage with the toolkit and broader IM approach, including collaboration with external partners; 2) simplicity was defined as the degree of ease with which the SDB IM toolkit and overall IM approach could be applied during outbreak responses; and 3) flexibility was defined as the extent to which the SDB IM toolkit and overarching IM approach could accommodate varying operational demands.

### Key informant interviews

Key informants from National Societies and the IFRC were purposively recruited following predefined criteria (**Table 2**). Recruitment began with an initial set of key informants and expanded through snowball sampling. For each KII, a tailored interview guide was used, with questions adapted to each informant’s areas of expertise (**Appendix**, **Supplemental material**, Table S1). Key informants were asked to reflect primarily on their most recent VHF outbreak experience, while drawing on prior outbreaks, where relevant, to provide additional context. Interviews were conducted with one facilitator and one notetaker, in English or French, during 45-minute sessions on Microsoft Teams (Microsoft 365).

**Table 2:** Inclusion and exclusion criteria

Criteria	Key informant interviews	Survey
Inclusion criteria	Key informants were eligible if they: were RCRC Movement volunteers, staff, or delegates; and had experience after the 2013–2015 West Africa EVD outbreak, in at least one of the following: SDB program development, or SDB training facilitation, or SDB field implementation.	Participants were eligible if they: were RCRC Movement volunteers, staff, or delegates; and had experience with SDB field implementation after the 2013–2015 West Africa EVD outbreak.
Exclusion criteria	Key informants were not eligible if they: had no direct involvement in SDB activities; only had relevant experience from the 2013–2015 West Africa EVD outbreak or earlier; or were unavailable to participate in an interview.	Participants were not eligible if they: had no direct SDB field experience; only had field experience from the 2013–2015 West Africa EVD outbreak or earlier; or provided incomplete survey responses.

Abbreviations: EVD, Ebola virus disease; RCRC, Red Cross and Red Crescent; SDB, Safe and Dignified Burial

Collection and analysis of interview data followed a modified approach to the Rapid Research Evaluation and Learning method, which focused on generating timely, actionable insights without a standalone analysis step (15,16). Following each KII, facilitators and notetakers reviewed notes to reconcile interpretations and document emerging themes on a shared and routinely updated synthesis sheet. Two authors then reviewed this synthesis sheet to identify overarching themes and iteratively refine subsequent interview guides to address remaining areas of inquiry.

### Survey

Building on preliminary findings from KII, an online survey was administered to further assess implementation of the SDB IM approach across the attributes of acceptability, simplicity and flexibility. Eligible participants were identified through collaboration with focal points from selected National Societies and IFRC, focusing on individuals involved in SDB field implementation after the 2013–2015 West Africa EVD outbreak (Table 2).

The survey was deployed using KoboCollect (KoboToolbox). It included both closed- and open-ended items, with skip logic to tailor questions based on participant experiences. Participants were encouraged to provide responses in relation to their most recent VHF outbreak experience. Survey data were analyzed using Microsoft Excel and R Studio (v2025.05.1+513).

### Ethical statement

As this evaluation was part of routine programming, formal ethical review was not sought. Data collection nevertheless followed ethical principles laid out in the Declaration of Helsinki (17). All key informants and survey participants were informed of study objectives, the voluntary nature of participation, and their ability to ask questions or withdraw. Data were anonymized, securely stored and deleted after analysis.

## Results

Eight KIIs were completed between December 11, 2024 and May 21, 2025 (**Table 3**). Despite some variation, perspectives were markedly consistent across key informants. As interviews progressed, insights began to converge toward saturation, supporting the robustness of the dataset.

**Table 3:** Key informants, affiliation and experience

ID	RCRC affiliation	Sex	Experience with SDBs
KII 1	IFRC headquarters (Geneva)	Female	Coordinated SDB activities during multiple VHF outbreaks.
KII 2	Canadian Red Cross	Female	Facilitated SDB training; deployed as IFRC delegate to multiple VHF outbreaks.
KII 3	Canadian Red Cross	Female	Developed SDB Emergency Response Unit concept and handbook; facilitated SDB training; deployed as IFRC delegate to multiple VHF outbreaks.
KII 4	British Red Cross	Female	Developed SDB IM toolkit; deployed as IFRC delegate to multiple VHF outbreaks.
KII 5	Guinea Red Cross	Male	Coordinated SDB activities during multiple VHF outbreaks.
KII 6	IFRC Regional Office for Africa (Nairobi)	Male	Remote SDB IM support; deployed as IFRC delegate to multiple VHF outbreaks.
KII 7	Democratic Republic of the Congo Red Cross	Male	Coordinated SDB activities during multiple VHF outbreaks.
KII 8	Uganda Red Cross	Male	Coordinated SDB activities during multiple VHF outbreaks.

Abbreviations: IFRC, International Federation of Red Cross and Red Crescent Societies; IM, information management; SDB, Safe and Dignified Burial; VHF, viral hemorrhagic fever

**Figure 1:**
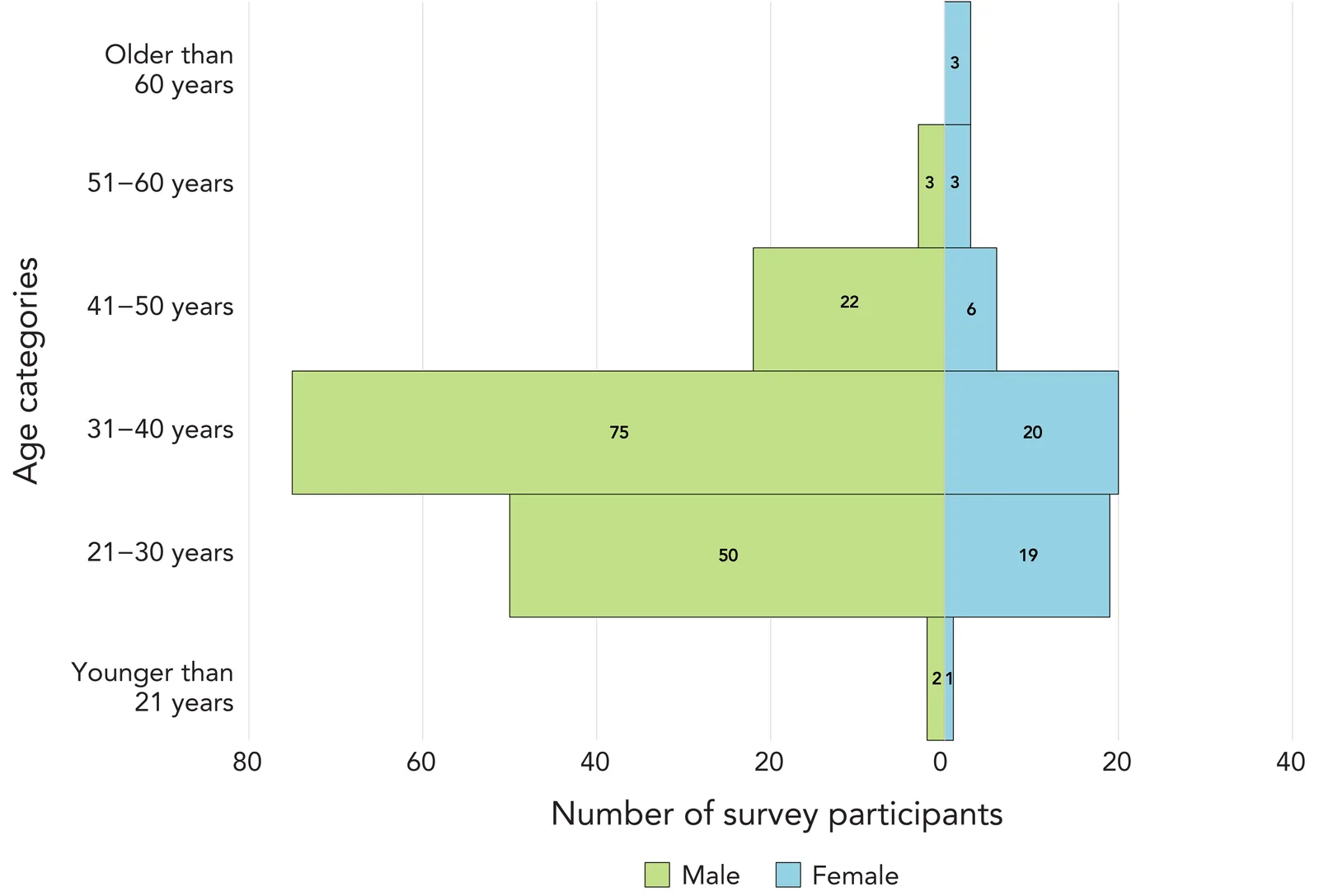
Age and sex of survey participants^a^ ^a^ n=208; 25.9% female and 74.1% male

**Figure 2:**
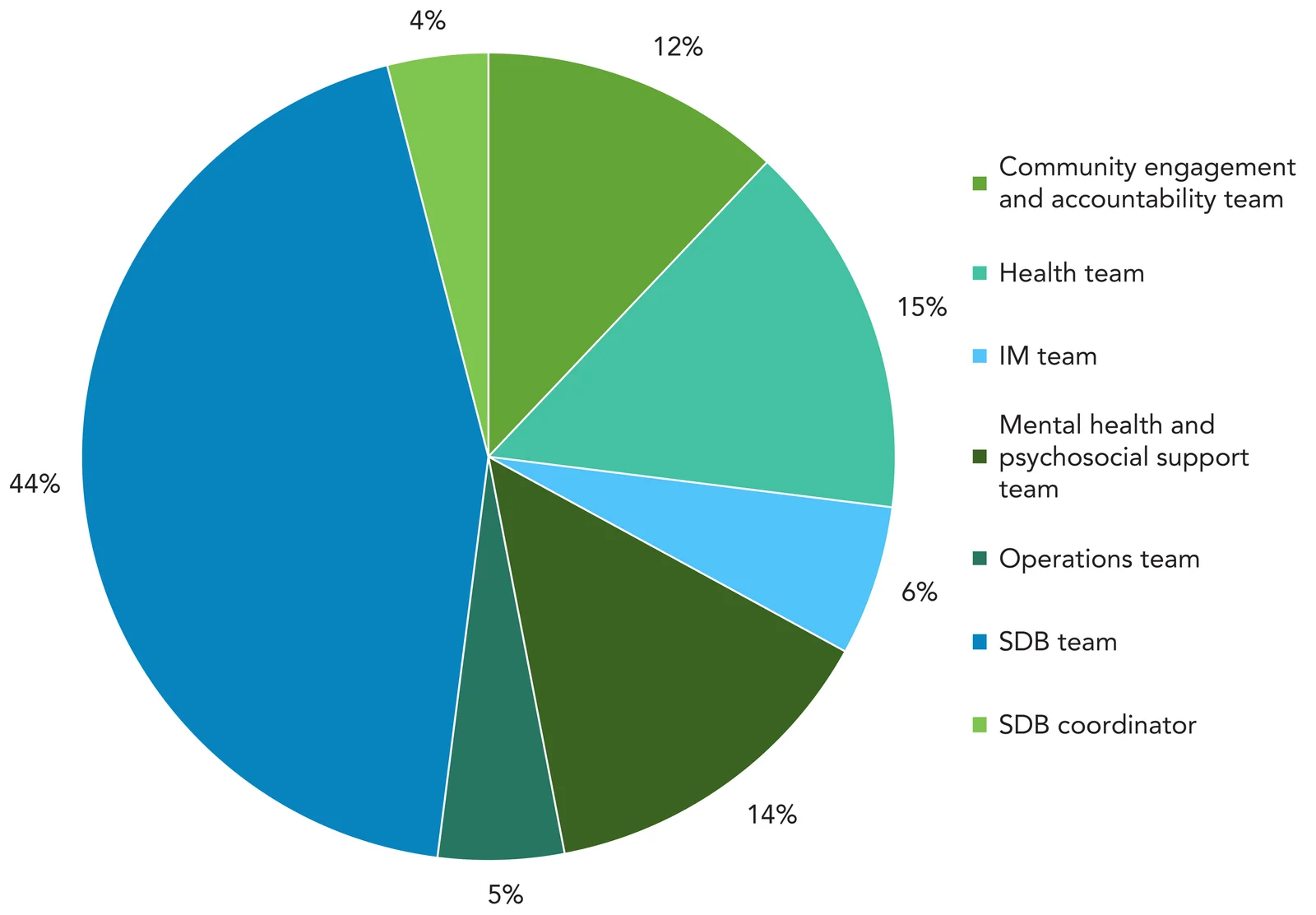
Survey participants, by role in most recent response Abbreviations: IM, information management; SDB, Safe and Dignified Burial

Between August 1, 2025–August 25, 2025, 256 survey responses were received. After excluding 48 records that did not meet inclusion criteria, 208 responses were retained for analysis (**Figure 1**, **Figure 2**, **Table 4**).

**Table 4:** Survey participant characteristics

Participant characteristics	n (%)
**SDB-related experience^a^**	
Completed SDB training	190 (91.3%)
Burial team supervisors	44 (21.2%)
Burial team members (supervisors and non-supervisors)	160 (76.9%)
Performed SDB data analysis and reporting	79 (38.0%)
Made operational decisions during VHF outbreaks (“decision-makers”)	67 (32.2%)
**Personnel category**	
RCRC volunteers	140 (67.3%)
RCRC staff	59 (28.4%)
RCRC delegates	6 (2.9%)
Not reported/missing	3 (1.4%)
**National Society Affiliation**	
British RC	6 (3.0%)
Canadian RC	2 (1.0%)
Democratic Republic of Congo RC	17 (8.0%)
French RC	2 (1.0%)
Guinea RC	152 (73.0%)
Tanzania RC	6 (3.0%)
Uganda RC	23 (11.0%)
Not reported/missing	0

Abbreviations: RC, Red Cross; RCRC, Red Cross and Red Crescent; SDB, Safe and Dignified Burial; VHF, viral hemorrhagic fever

^a^ Participants reported Safe and Dignified Burial preparedness or response experience across 11 countries: Burundi, Democratic Republic of the Congo, Equatorial Guinea, Gabon, Guinea, Kenya, Rwanda, Sierra Leone, Sudan, Tanzania and Uganda

### Acceptability

Across contexts, the overarching approach to SDB IM was generally well accepted. Following training, 75.0% of burial team supervisors felt “very” or “mostly confident” collecting SDB data (Appendix, Supplemental material, Table S2). Despite the sensitivities of collecting information from bereaved families, 66.3% of burial team members were “very” or “mostly comfortable” fulfilling this role, though additional training on community engagement was commonly requested in free-text responses. Among decision-makers, 85.1% found SDB reporting outputs to be “very” or “mostly useful” and 82.1% said these reports “consistently” or “frequently” impacted their decision-making.

Key informants also described multifaceted and mutually beneficial relationships between National Societies and local authorities from different outbreak contexts. For instance, Ministry of Health (MoH) representatives attended RCRC-led SDB training and MoH surveillance alerts helped inform the day-to-day deployment of burial teams. Where available, burial team members followed MoH testing protocols for community deaths, with positive results potentially initiating a cascade of MoH-led response activities like contact tracing and ring vaccination. Furthermore, SDB data summaries were routinely shared with local authorities and other humanitarian partners at interagency coordination meetings.

Despite these strengths, awareness of the SDB IM toolkit was notably uneven across RCRC personnel. Although some key informants—and 15.2% of survey participants involved in analysis and reporting—indicated no prior awareness of the toolkit, documents shared by National Societies showed that templates from the toolkit were nevertheless being used, suggesting indirect or partial dissemination without accompanying guidance. Key informants further noted that the value of SDB IM was often not clearly communicated to National Society leadership and RCRC teams outside SDB operations. Heavy workloads for burial teams were also noted, alongside frustration with terminology that classified SDB outcomes as “successful” or “failed.” Key informants indicated that such language did not adequately reflect the complex social and operational realities of burial activities, particularly in situations involving community resistance or incomplete burials despite appropriate efforts and could negatively affect team motivation.

### Simplicity

Key informants described clear SDB IM workflows and a solid foundation of SDB training to prepare them for field implementation. They further commended the toolkit’s ability to facilitate a phased approach to implementation; for instance, starting with a limited set of essential data points before expanding to more detailed reporting as responses progressed. Survey findings were also aligned, with 77.5% of burial team supervisors indicating that SDB data collection forms were “very” or “mostly easy” to understand, and 85.0% of decision-makers noting that SDB reports were “very” or “mostly clear” in providing actionable intelligence.

In terms of challenges, key informants familiar with the SDB IM toolkit reported that the accompanying guidance documents were too text-heavy, too detailed and overly prescriptive without taking local operational environments into account. These challenges were commonly reported as a barrier to field-level utilization. Participants further noted the need for more IM-specific training, hands-on practice with data collection and regular refresher training to sustain readiness over time.

### Flexibility

The SDB IM approach demonstrated versatility across outbreak settings with varying linguistic, cultural and operational needs. Key informants noted that the toolkit supported both paper-based and mobile data collection, enabling National Societies to adapt activities to differing levels of human resource capacity and connectivity. They also appreciated the availability of key documents in French and English, reflected in survey findings where 97.4% of participants reported receiving training in an appropriate language.

The flexibility of the SDB IM approach was reportedly constrained by limited data analysis and reporting capacity within National Societies. For instance, while 72.2% of survey participants involved in SDB analysis and reporting received pre-developed reporting templates, 34.2% were only able to adapt these templates to local contexts “with difficulty” or “not at all.” Key informants and survey participants also commonly highlighted opportunities to strengthen IM capacity among National Society personnel with the aim of reducing reliance on technical support from the IFRC.

## Discussion

Acceptability of the SDB IM approach was broadly positive, but several gaps limited its wider adoption. Burial teams routinely gathered essential information, often in close collaboration with local authorities, feeding into established IM workflows that supported data-informed decision-making during outbreaks; however, uneven awareness of the SDB IM toolkit among RCRC personnel, along with gaps in communicating the strategic value of IM and the heavy operational demands placed on burial teams, reduced overall acceptability.

Simplicity of the IM approach was viewed favourably, though several practical challenges limited ease of implementation. Clearly defined workflows enabled teams to carry out core IM tasks, while the toolkit’s phased approach allowed activities to start simply and expand as response needs evolved; however, overly detailed guidance documents, limited IM-specific training and insufficient opportunities for practice with hands-on data collection undermined simplicity.

Flexibility of the SDB IM approach was evident across outbreak settings with differing linguistic, cultural and operational needs. The availability of both paper-based and mobile data collection tools, along with key documents in different languages, enabled National Societies to adapt implementation to local capacity and connectivity constraints; however, limited data analysis and reporting capacity within some National Societies constrained the flexibility of operations, highlighting the need to further strengthen IM capacity to support greater local leadership across the IM workflow.

### Strengths and limitations

This evaluation benefited from engagement with a diverse group of RCRC Movement personnel with extensive experience across SDB responses, enabling triangulation of findings from multiple sources; however, several limitations should be considered. Key informants were purposively selected, meaning some perspectives may have been underrepresented. Survey responses were unevenly distributed across National Societies, with a large proportion originating from the Guinea Red Cross. This reflects differential response rates rather than differences in sampling and sensitivity analyses, which excluded these responses, yielded similar results. Recall bias may have affected responses due to the long time elapsed since some outbreak experiences. Researcher bias was also possible, given the study team’s affiliation with the RCRC Movement, although the use of standardized tools and team-based analysis helped mitigate this risk. Finally, the evaluation was not designed or powered to assess differences by sex or other demographic characteristics, which may influence operational roles and experiences during outbreak responses.

### Recommendations

Based on these findings, practical, implementation-focused recommendations were developed to strengthen the SDB IM approach. First, coordination of SDB IM could be strengthened by promoting regular communication across RCRC Movement entities, providing structured orientations on the SDB IM toolkit for National Society personnel, and implementing pre- and post-deployment briefings for international delegates supporting SDB IM. Second, awareness of the SDB IM toolkit could be increased by promoting consistent use of the toolkit across responses, demonstrating the value of data-informed decision-making during outbreaks, and supporting leaders to reinforce the strategic importance of SDB IM. Third, greater localization of SDB IM implementation could be supported by increasing involvement of National Society personnel across all stages of SDB IM, enabling contextual adaptation of tools and workflows while maintaining core standards, and promoting mentorship with stepwise handover of SDB IM functions to National Society personnel. Finally, SDB IM training could be expanded by allocating dedicated time for SDB IM-specific competencies and practical data collection exercises, and providing regular refresher training to sustain operational readiness over time.

## Conclusion

While gaps remain, this evaluation highlights the role of the SDB IM approach in VHF outbreak response across the RCRC Movement. Strengthening coordination, increasing awareness of the toolkit, expanding IM capacity and supporting greater local ownership may further enhance readiness for future responses.

## Appendix

Supplemental material is available upon request to the author: doug.k.lau@gmail.com

Table S1: Key informant interview question guide

Table S2: Survey results
